# The relationships among nurses’ spiritual health, sleep quality, and stress and the factors influencing stress during the late global COVID-19 pandemic: A cross- sectional study

**DOI:** 10.1371/journal.pone.0323164

**Published:** 2025-05-12

**Authors:** Yueh-E. Lin, Li-Yu Chien, Mei-Lien Hu

**Affiliations:** 1 Department of Nursing, Linkou Chang Gung Memorial Hospital, Taoyuan, Taiwan; 2 Department of Nursing, Chang Gung University of Science and Technology, Taoyuan, Taiwan; GCS Medical College, INDIA

## Abstract

**Background:**

COVID-19 has had a significant impact on healthcare workers. Although several studies have looked at the pandemic’s physical and mental effects on nurses, little has been done to investigate their spiritual health and its relationship to stress and sleep quality during the late pandemic.

**Purpose:**

This study sought to fill a knowledge gap in the literature about the relationships between nurses’ reported stress, sleep quality, and spiritual health during the late COVID-19 epidemic.

**Methods:**

A cross-sectional study using purposive sampling was performed out in a medical center in Taiwan. A total of 376 nurses participated. The Perceived Stress Scale, Pittsburgh Sleep Quality Index, and Spiritual Health Scale-Short Form were used for assessing nurses’ stress levels, sleep quality, and spiritual health.

**Results:**

The results showed that the mean perceived stress score was 1.80 ± 0.50 (out of 4), the sleep quality score was 8.17 ± 3.29 (out of 21), and the mean spiritual health score was 3.66 ± 0.59 points (out of 5). Although 77.1% of the nurses in this study experienced sleep disorders (PSQI > 5), they had better sleep quality during the COVID-19 pandemic than those in other countries. Perceived stress, sleep quality, and spiritual health were significantly correlated. Nurses with support from their friends and family and hospital during the COVID-19 pandemic had lower perceived stress and higher sleep quality and spiritual health scores than their peers (*p* < .05). Age, work experience, sleep quality, and spiritual health were predictors of perceived stress in nurses during the late COVID-19 pandemic (*F* = 20.19, *p *< .001) and could explain 30.6% of the variation.

**Conclusions:**

Spiritual health is correlated with the nurses’ stress levels. Despite providing extrinsic support, we encourage nursing management to pay attention to nurses’ spiritual needs and implement psychological education programs to help frontline nurses navigate ever-changing and discerning healthcare environments.

## Introduction

The 2019 novel coronavirus disease (COVID-19) pandemic has caused unprecedented mass panic among the global population and led to enormous challenges for clinical staff. Frontline nurses in clinical care have the most contact with patients. Although the COVID-19 pandemic has highlighted the special dedication of nurses, it has also had seriously physically and psychosocially impacted them.

During the pandemic, 48% of nursing professionals showed signs of depression, 52% exhibited anxiety, 52% displayed indications of stress, 75% had sleep disorders, and 68% reported insomnia [1]. Although the world has entered a post pandemic era, there has been an increase in nurses’ burnout and turnover [2], which has exacerbated nursing shortages and patient care. Medical institutions must learn about the pandemic and be prepared at all times to face the challenge of the next unknown major infectious disease. Therefore, looking back at the impacts on and needs of nurses during the pandemic may help provide better support for nurses and adequately prepare for the next challenge.

A large body of research has examined the effects of stress related to the COVID-19 pandemic on nurses. The literature shows that nurses experienced greater stress than other medical staff during the pandemic [3], and that stress is negatively correlated with clinical performance in nurses [4]. Many studies have revealed that nurses’ work stressors during the pandemic are mostly derived from heavy care burdens [3,5], inadequate personal protective equipment [5–7], fear of becoming infected [5,7] and isolation from society [6,8]. The impacts of the pandemic and the lack of support from family and society may also cause immense physical and mental stress in frontline nurses, resulting in a decline in sleep quality [9–11].

Although many studies have examined the impact of the COVID-19 pandemic on nurses’ sleep quality. There is no consensus on this aspect.. Approximately 75.0% of all nurses in Brazil had sleep disorders [1], with 73.3% in Japan [12], and 71.4% in Italy [13] reporting sleep issues. However, only 26.4% of nurses presented with sleep disorders in a Brazilian study [14] and 52.8% in Chinese study, respectively [15], and 82.4% of nurses reported no change in their sleep quality among Norwegian nurses [16]. Although there is no consensus regarding the number of nurses whose sleep quality was affected by the COVID-19 pandemic, it has been proven that the poor sleep quality during the pandemic is positively correlated with the degree of stress, anxiety, fatigue, and fear of COVID‐19 among nurses [12,15].

Spirituality is a dimension of well-being that should be included in holistic health and has attracted much attention in the health-related and nursing sciences. During an unknown pandemic, nurses often struggle with patient care and self-protection. Moral distress can lead to spiritual exhaustion [17]. However, few studies have examined nurse’s spiritual health during the COVID-19 pandemic. Spirituality refers to internal motives that give meaning and hope to an individual’s life and an essence that gives meaning and purpose to their individual’s existence [18]. People with high spiritual health have better emotional, social, physical, and mental resilience [19], and spiritual health has been shown to positively impact professional commitment and care among nurses [20,21]. It is evident that the spiritual well-being of advance practice nurses has been significantly impacted during the COVID-19 pandemic [22], and spirituality is a common strategy used by nurses when coping with stress and burnout [23]. However, relatively few studies have examined nurses’ spiritual health and its relationship with stress during the global pandemic. Accordingly, the current study aimed to examine the correlation between perceived stress, sleep quality, and spiritual health in nurses, as well as factors influencing stress during the late global pandemic.

## Materials and methods

### Study design and samples

This cross-sectional study was conducted at a medical center in northern Taiwan. A cross-sectional study design was selected because it is the best method to determine the prevalence of health outcomes, and examine the association between multiple exposures and outcomes [24]. We collected and analyzed data from frontline nurses at a single time point. Convenience sampling was employed, and frontline nurses were recruited to participate in this study from June 2022 to December 2022. Although COVID-19 was initially discovered in late 2019, the World Health Organization (WHO) did not declare COVID-19 a global health emergency until March 2020 and the public health emergency declarations ended in May 2023 [25], the period of data collection was identified as the late pandemic period.

The inclusion criteria were frontline nurses working full-time in general wards and intensive care units in the medical center during the pandemic and available at the time of data collection. Nurses with no previously reported characteristics were excluded from the study. G*Power 3.1.9.4 software was used for sample size estimation. Multiple regression was employed as the statistical method, with a significance level (*α*) of 0.05 and power of 0.8. An effect size of 0.15 was used as a reference[24], and the minimum sample size needed was 217. A total of 400 frontline nurses were invited to participate in this study, and 376 participants completed the survey. The response rate of valid questionnaires was 94.0%.

### Ethical considerations

This study was approved by the Institutional Review Board of Chang Gung Memorial Hospital (approval no. 202101826A3C101) prior to data collection. The data collected in this study were used only for this research and all participants’ information was anonymized to ensure confidentiality. The nurses were informed of their right to refuse to participate in the study or withdraw at any time before completing the questionnaires and that there would be no consequences for doing so. During the case collection process, the interviewees can choose to participate voluntarily or not, and the rights and interests will not be affected.

### Measurements/instruments

#### Demographic characteristics.

The demographic characteristics collected in this study included gender, age, marital status, education level, years of working experience, working unit, religion, experience of caring for COVID-19 patients, willingness to work in dedicated COVID-19 wards, whether the participants had children, support from friends and family, and support from the hospital during the COVID-19 pandemic.

#### Perceived Stress Scale (PSS).

The Perceived Stress Scale (PSS) [26] was used to determine the degree of stress perceived by nurses during the COVID-19 pandemic. The Taiwanese version of PSS was translated by Chu & Kao (2005). The scale has good internal consistency (Cronbach’s α = .84–.86) and test–retest reliability (*r* = .85) [27]. This 5-point Likert scale with 14 questions is a self-reported tool to measure an individual’s thoughts on lifestyle stress in the preceding month. The score for each question ranges from 0 to 4, with never = 0, occasionally = 1, sometimes = 2, often = 3 and always = 4. The total score ranges from 0 to 56 points with higher scores indicating greater perceived stress.

#### Pittsburgh Sleep Quality Index (PSQI).

The Pittsburgh Sleep Quality Index (PSQI) [28] was used to measure sleep quality. This self-rated questionnaire assesses sleep quality and disturbances over a 1-month time interval. The Taiwanese version was translated by Tsai et al. (2005). The scale’s sensitivity and specificity were 98% and 55%, respectively, and Cronbach’s α of.82–.83 [29]. The scale contains 19 questions, and its contents include subjective sleep quality, sleep latency, sleep duration, habitual sleep efficiency, sleep disturbances, sleeping medication use, and daytime dysfunction. The score for each item ranges from 0 to 3 points (0 = no difficulty, 3 = severe difficulty), and the total score ranges from 0 to 21 points with higher scores denoting poorer sleep quality. A total PSQI score of 5 points was used as a cutoff [29–32]. in which a total score ≦ 5 indicated good sleep quality and a total score > 5 indicated poor sleep quality [28].

#### Spiritual Health Scale-Short Form (SHS-SF).

The Spiritual Health Scale-Short Form (SHS-SF) [33] was employed to assess spiritual health this study. This Taiwanese scale measures nurses’ spiritual health and is divided into five subscales: connection to others, meaning derived from living, transcendence, religious attachment, and self-understanding. Responses to the scale’s 24 questions are recorded on a Likert scale ranging from 1 to 5 points, with 1 representing “extremely disagree” and 5 representing “extremely agree.” The total score ranges from 24 to 120 points with higher scores indicating better spiritual health. The construct reliability of the original scale was between 0.86 and 0.90, and the average variance extracted ranged from 0.60 to 0.69, which shows good convergent validity [33]. The Cronbach’s *α* of the various subscales ranged from 0.86 to 0.92.

#### Data collection/procedure.

The data were collected from June 2022 to December 2022. As Taiwan has downgraded the legal status of COVID-19 to a less serious disease since May 2023, the duration of data collection can be identified as the late pandemic period. After the study was approved by the Institutional Review Board (IRB), the researcher went to the wards or intensive care units during a pre-shift meeting (i.e., a brief meeting held before the start of each work shift) to explain the study objectives to nurses and invited them to participate. Informed consent forms and questionnaires were left for nurses to read and complete. After completion, participants were asked to return the questionnaires by placing them in a sealed envelope left by the researcher. The advantage of this data collection method is that nurses can complete the questionnaire anonymously and have sufficient time to answer the questions without fear of prejudice.

#### Data analysis.

The IBM SPSS Statistics 26.0 program was used for data analysis. Means and standard deviations were used for the stress, sleep, and spiritual health scores of nurses during the COVID-19 outbreak. An independent *t* test and one-way ANOVA were used to compare the differences in stress, sleep, and spiritual health scores between groups with different characteristics, a Pearson correlation coefficient was employed to examine the relationship between stress, sleep, and spiritual health, and multiple linear regression analysis was used to analyze the influencing factors of stress among nurses during the COVID-19 pandemic.

## Results

### Demographic characteristics of participants

A total of 376 nurses completed the questionnaire, with a response rate of 94.0%. Table 1 lists the demographic characteristics of the frontline nurses in this study. The mean age of the participating nurses was 31.52 ± 7.97 years, with an average working experience of 8.70 ± 7.77 years. Approximately 96.0% of the nurses were women, 55.6% had religious beliefs, 85.9% had graduated from university, 72.1% were single, 78.5% did not have children, and 32.7% were the breadwinners of their families. A total of 20.2% of the nurses were concerned about their living arrangements during the late COVID-19 pandemic, 94.9% perceived that they received support from friends and family, and 89.9% felt that they received sufficient support from the hospital. Of the 376 nurses, 160 (42.6%) had experience caring for COVID-19 patients and 122 (76.3%) had volunteered to be deployed to dedicated COVID-19 wards.

**Table 1:** Demographic characteristics of participant nurses. (n = 376).

Characteristic	Mean (SD)	*n* (%)
Age (years)	31.52 (7.97)	
Working experience (years)	8.70 (7.77)	
Sex		
Men		15 (4.0)
Women		361 (96.0)
Religious beliefs		
Yes	–	209 (55.6)
No	–	167 (44.4)
Nursing education		
Associate degree	–	33 (8.8)
Bachelor’s degree	–	323 (85.9)
Master’s degree		20 (5.3)
Marital status		
Single	–	271 (72.1)
Married	–	105 (27.9)
Having children		
Yes		81 (21.5)
No		295 (78.5)
Working unit		
Ward		267 (71.0)
Intensive care unit		109 (29.0)
Breadwinner of the family		
Yes		123 (32.7)
No		253 (67.3)
Concern about living arrangements during the COVID-19 pandemic		
Yes		76 (20.2)
No		300 (79.8)
Supported by family and friends		
Yes		357 (94.9)
No		19 (5.1)
Supported by the hospital		
Yes		338 (89.9)
No		38 (10.1)
Experience caring for COVID-19 patients		
Yes		160 (42.6)
No		216 (57.4)
Volunteered to be deployed to COVID-19 dedicated wards (n = 160)		
Yes		122 (76.3)
No		38 (23.8)

Note. SD: Standard deviation; COVID-19: 2019 coronavirus disease.

### Perceived stress, sleep quality and spiritual health

Table 2 presents the results for perceived stress, sleep quality, and spiritual health of nurses during the late COVID-19 pandemic. The mean perceived stress score was 1.80 ± 0.50 (out of 4) with a total score of 25.16 ± 6.93 (out of 56 points), the sleep quality score was 8.17 ± 3.29 (out of 21), and the mean spiritual health score was 3.66 ± 0.59 points (out of 5 points). The mean daily sleep duration was 6.89 ± 1.32 hours, and 77.1% of nurses perceived sleep disorders (PSQI > 5 points).

**Table 2:** Perceived stress, sleep quality, and spiritual health of participant nurses (n = 376).

Variables	Mean (SD)	n (%)
Perceived stress mean score (0–4)	1.80 (0.50)	
Perceived stress total score (0–56)	25.16 (6.93)	
PSQI		
Sleep duration mean score (hrs.)	6.89 (1.32)	
Total sleep score (0–21)	8.17 (3.29)	
Sleep disorder		
Yes (> 5)		290 (77.1)
No (≤ 5)		86 (22.9)
Spiritual health mean score (1–5)	3.66 (0.59)	
Connection with others	4.01 (0.75)	
Meaning derived from living	3.80 (0.72)	
Transcendence	3.60 (0.69)	
Religious attachment	3.18 (0.98)	
Self-understanding	3.66 (0.75)	
Total spiritual health score (24–120)	87.80 (14.12)	

Note. SD: Standard deviation; PSQI: Pittsburgh Sleep Quality Index.

Table 3 shows the analysis of the differences in perceived stress, total sleep score, and spiritual health score. Nurses with religious beliefs showed better spiritual health than nurses without religious beliefs (*p* = .41). Nurses who were the family’s breadwinner had poorer sleep quality than those who were not the breadwinner (*p* = .010). Nurses who did not volunteer to be deployed in COVID-19 wards had poorer sleep quality than those who volunteered to be deployed in dedicated wards (*p* < .001). Nurses with COVID-19 care experience had lower perceived stress than nurses without COVID-19 care experience (*p* = .045). Furthermore, nurses who received support from friends and family during the COVID-19 pandemic had lower stress (*p *= .017), better sleep quality (*p* = .012), and better spiritual health (*p* < .001) than their counterparts. Similarly, nurses with sufficient perceived support from the hospital during the pandemic had lower stress (*p* = .006), better sleep quality (*p* = .010), and better spiritual health (*p* = 006). However, there were no significant differences in perceived stress, sleep quality, and spiritual health scores by education level, marital status, work unit, or concerns about living arrangements during the epidemic.

**Table 3:** Comparison of perceived stress, sleep quality, and spiritual health with demographic characteristics (n = 376).

Variables	Perceived stress (0–4)		Sleep quality (0–21)		Spiritual health (1–5)	
	Mean (SD)	*p*	Mean (SD)	*p*	Mean (SD)	*p*
Religious beliefs		.706		.829		.041^*^
Yes	1.81 (0.50)		8.21 (3.38)		3.71 (0.62)	
No	1.79 (0.48)		8.13 (3.19)		3.59 (0.54)	
Nursing education		.440		.947		.173
Associate’s degree	1.73 (.63)		8.21 (3.93)		3.67 (.78)	
Bachelor’s degree	1.81 (.49)		8.15 (3.27)		3.64 (.55)	
Master’s degree	1.70 (.34)		8.40 (2.62)		3.90 (.76)	
Marital status		.092		.386		.136
Single	1.83 (0.49)		8.10 (3.38)		3.63 (0.59)	
Married	1.73 (0.52)		8.43 (3.07)		3.73 (0.58)	
Having children		.135		.621		.147
Yes	1.72 (.52)		8.33 (3.12)		3.74 (.60)	
No	1.82 (.49)		8.13 (3.35)		3.64 (.58)	
Working unit		.805		.913		.364
Ward	1.80 (.47)		8.16 (3.42)		3.64 (.59)	
Intensive care unit	1.79 (.55)		8.20 (2.99)		3.70 (.59)	
Breadwinner of the family		.946		.010^*^		.799
Yes	1.79 (.50)		8.80 (3.26)		3.66 (.52)	
No	1.80 (.49)		7.87 (3.28)		3.65 (.62)	
Concern about living arrangements during the COVID-19 pandemic		.159		.151		.328
Yes	1.87 (.50)		8.66 (3.56)		3.60 (.70)	
No	1.78 (.49)		8.05 (3.22)		3.67 (.56)	
Experience caring for COVID-19 patients		.045^*^		.089		.986
Yes	1.74 (.50)		7.84 (3.18)		3.66 (.54)	
No	1.84 (.49)		8.42 (3.37)		3.66 (.63)	
Volunteered to be deployed to COVID-19 wards (n = 160)		.584		<.001^***^		.577
Yes	1.73 (0.50)		7.34 (3.06)		3.67 (0.53)	
No	1.78 (0.49)		9.45 (3.04)		3.62 (0.57)	
Supported by family and friends		.017^*^		.012^*^		<.001^***^
Yes	1.78 (.49)		8.08 (3.22)		3.69 (.57)	
No	2.08 (.57)		10.00 (4.10)		3.12 (.72)	
Supported by the hospital		.006^**^		.010^*^		.006^**^
Yes	1.77 (.49)		8.03 (3.23)		3.69 (.58)	
No	2.01 (0.53)		9.47 (3.65)		3.41 (.60)	

Note.

*p < .05;

**p < .01;

***p < .001; SD: Standard deviation.

Table 4 shows the results of Pearson’s correlation between spiritual health, perceived stress, sleep quality, and continuous demographic variables including age and working experience of nurses during the pandemic. The higher the perceived stress, the poorer the sleep quality (*r* = .384, *p* < .01), the higher the perceived stress, the lower the spiritual health (*r* = -.388, *p* < .01), and the poorer the sleep quality, the lower the spiritual health (*r* = -.217, *p* < .01). In addition, nurses with more years of work experience had lower perceived stress (*r* = -.165, *p *< .05) but had poorer sleep quality (*r* = .106, *p* < .05). There was no significant correlation between work experience and spiritual health (*p *> .05).

**Table 4:** Correlation between spiritual health, perceived stress, sleep quality, and continuous demographic variables including age and working experience of nurses during the late COVID-19 pandemic (n = 376).

Variables	Working experience (years)	Perceived stress	Sleep quality	Spiritual health
Age (years)	.949^**^	-.141^*^	.091	.018
Working experience (years)		-.165^*^	.106^*^	-.027
Perceived stress		–	.384^**^	-.388^**^
Sleep quality		–	–	-.217^**^

Note.

*p < .05;

**p < .01; COVID-19: Coronavirus Disease 2019.

Table 5 shows a multiple regression analysis of predictors of perceived stress in the nurses. The results showed that age (*β *= .331, *p *< .019), work experience (*β *= -.528, *p *< .001), sleep quality (*β *= .322, *p *< .001), and spiritual health (*β *= -.332, *p *< .001) were predictors of perceived stress in nurses (*F *= 20.19, *p *< .001) during the late COVID-19 pandemic. It could explain 30.6% of the variation (*R*^*2* ^= .306).

**Table 5:** Factors affecting perceived stress of nurses during the late COVID-19 pandemic. (n = 376).

Variables	Beta	SE	*t*	*p*	95% CI		VIF
					lower	upper	
(Constant)		.256	8.39	<.001^***^	1.65	2.66	
Age (years)	.331	.009	2.36	.019^*^	.003	.038	10.393
Working experience (years)	-.528	.009	-3.76	<.001^***^	-.051	-.016	10.423
Breadwinner	.032	.050	.68	.499	-.064	.131	1.172
Experience of caring for COVID-19 patients	-.063	.044	-1.44	.150	-.150	.023	1.018
Supported from family and friends	-.008	.105	-.17	.864	-.225	.188	1.144
Supported from the hospital	-.036	.075	-.78	.433	-.208	.089	1.119
Sleep quality	.322	.007	7.06	<.001^***^	.035	.062	1.103
Spiritual health	-.332	.039	-7.22	<.001^***^	-.355	-.203	1.115

Note.

*p < .05;

***p < .001; R^2^:.306; F: 20.19; COVID-19: Coronavirus Disease 2019; SE: Standard Error; CI: Confidence Interval.

## Discussion

### Perceived stress

This study found that the mean perceived stress score of nurses during the COVID-19 pandemic was 25.16 ± 6.93 (out of 56 points). A study by Leng et al. [5] showed that the mean perceived stress score of nurses during the pandemic was 19.33 ± 7.21; thus, the nurses in our study had higher perceived stress. However, further analysis of the backgrounds of the participants in these two studies revealed that the participants in Leng et al. [5] were nurses who cared for COVID-19 patients in an intensive care unit, whereas our study recruited frontline nurses with and without experience in caring for COVID-19 confirmed cases. Nurses who cared for confirmed cases were experienced in the actual care process and the hospital provided comprehensive protective measures when they cared for confirmed patients. Consequently, their perceived stress levels were lower. This argument can be proven by the results of this study; that is, nurses who received sufficient support from the hospital had significantly lower perceived stress scores than those who reported no support (*p* < .01). Another reason could be that the actual care experience helps decrease uncertainty about COVID-19. The multiple linear regression analysis results also validated that work experience is an influencing factor that predicts perceived stress. According to a survey by Huang et al. [34] facing emerging and highly contagious diseases in Taiwan will increase the stress of medical staff. In the early stages, the stress of medical staff will be affected by the progress of the epidemic. The lack of supplies and information may put medical staff under greater pressure.

In addition, this study found that nurses who received support from friends, family, and the hospital reported lower perceived stress. Previous studies have shown that a shortage of protective equipment [5,7] and isolation from friends, family, and society [6,8] can result in stress for nurses during the COVID-19 pandemic. An integrative review [35] also concluded that loss of life and lack of supportive leadership contributed to symptoms of anxiety, stress, depression, and moral distress in nurses during the global pandemic. The ability to discuss their stress or work experiences with loved ones may help nurses reduce the psychological burden of suppressed emotions during the late pandemic.

Furthermore, support from the hospital that contributed to decreased stress included the provision of sufficient personal protective equipment and other supplies such as monetary pay. In response to the impact of the global pandemic, the Taiwanese government announced a special act for COVID-19 and provided frontline nurses who cared for COVID-19 patients with NT$10,000 (US$ 334.43) for each eight-hour shift worked [36]. For individual employees, pay is an important reward for their work. It has also been shown that nurses’ perceptions of organizational support and monetary pay satisfaction were positively related to their job satisfaction and negatively correlated with turnover intention during the COVID‐19 pandemic [37]. This indicates that nurses require both intrinsic and extrinsic support to combat the impacts of a pandemic. Sufficient extrinsic support from hospitals and intrinsic support from friends and family during the late pandemic have allowed nurses to devote themselves to clinical care with peace of mind. It was conducted in 2022, and medical staff were more affected than Leng et al. [5], Galehdar et al. [6], and Arnetz et al. [8] in the early stage of the epidemic in 2020.

### Sleep quality

The results of this study showed that 77.1% of nurses had sleep disorders (PSQI > 5) with a mean score of 6.89 ± 1.32 during the late COVID-19 pandemic, and sleep quality was an influencing factor for perceived stress. Other studies conducted during the COVID-19 pandemic have shown that the prevalence of sleep disorders among nurses ranged from 26.4% to 75.0% [1,12–15]. Although there is no consensus regarding sleep disorders among nurses during the COVID-19 pandemic, our results are similar to those of studies conducted in Brazil [1], Japan [12] and Italy [13]. Our study also proved that nurses’ poor sleep quality during the late pandemic was significantly correlated with the degree of stress [12,15].

Compared with the average sleep quality score of 9.85 ± 2.72 in the United States [9] and 8.48 ± 3.63 in China [11], the nurses in this study had a lower mean PSQI score (6.89 ± 1.32), indicating better sleep quality during the COVID-19 pandemic. However, this may be due to the different survey periods used in the studies. Studies in the United States and China were conducted during the early COVID-19 pandemic, whereas this study’s data were collected during the late global pandemic in 2022. Notably, nurses during the late pandemic may have learned how to react to and cope with the pandemic. Consequently, the sleep quality of the nurses in this study was relatively less affected by the COVID-19 pandemic. In addition, the nurses in this study had a similar prevalence of sleep disorders compared to a Korean study conducted before the COVID-19 pandemic in which 79.8% of nurses reported sleep disorders [38]. This might also indicate that nurses’ sleep quality is always a concern and that nurse leaders and executives should consider measures to improve it.

### Spiritual health

Regarding spiritual health, our study found that the mean spiritual health score of nurses during the COVID-19 pandemic was 3.66 ± 0.59. Lee et al. [39] investigated the physical and mental status of nurses who served as frontline caregivers during the COVID-19 epidemic in Taiwan. The average spiritual health was 3.79 ± 0.58, which was not much different from this study, but it was proposed that spiritual health provides the meaning of life. Since few studies have examined the spiritual health of nurses during the late pandemic, there are no available study results for comparison. Among the few studies that examined spiritual health in nurses, Chiang et al. [20] showed that the spiritual health subscale scores of connections to others, meaning derived from living, transcendence, religious attachment, and self-understanding were 4.35, 4.09, 3.78, 3.75, and 3.82, respectively, which were higher than the scores revealed in this study (4.01, 3.80, 3.60, 3.18, and 3.66, respectively). Hence, nurses’ spiritual health decreased during the COVID-19 pandemic, indicating that the pandemic negatively impacted nurses’ spiritual health.

Furthermore, this study showed that spiritual health was an influencing factor for perceived stress among nurses during the COVID-19 pandemic. Consistently ignoring professional caregivers’ needs can seriously affect their emotional and spiritual health. Therefore, in addition to providing equipment and mental support from friends and family, satisfying the spiritual needs of nurses helped decrease perceived stress during the late pandemic. As previously noted, nurses commonly use spirituality to cope with stress and burnout [23]. A systematic review also revealed that spiritual education significantly decreases nurses overall stress of nurses [40]. It has been suggested [17] that the first step toward spiritual and moral resilience is self-awareness. Nurses can be trained to identify symptoms of spiritual fatigue and adopt adaptive coping strategies to reconnect with the self and meaning of life. Working in a rapidly changing healthcare environment is always stressful, and frontline nurses must be prepared to cope with this ever-changing and stressful environment. We recommend that nursing managers provide spiritual health education to nurses, especially when they face enormous challenges such as an epidemic. This will help frontline nurses cope with the challenges posed by major infectious diseases.

### Limitations

This study has several limitations. First, the long-term impact of spiritual health on stress could not be determined because this study used a cross-sectional methodology. Second, this study only collected data from a single medical center; thus, the generalizability of the results is limited. Furthermore, the survey period of this study was 2022, a period in which many countries experienced several peaks in outbreaks during the late pandemic. Therefore, the results of this study may only reflect the stress, sleep quality, and spiritual health of nurses during the “late pandemic era.” Further studies are required to determine how spiritual health affects nurses’ stress levels and health outcomes. Third, the purposive sampling technique may introduce selection bias, which may affect the generalizability of the research results. In addition, factors such as shift patterns, working hours, and direct contact with COVID-19 patients that may affect stress and sleep quality were not collected. It is recommended that further discussion be conducted in the future.

## Conclusions

In the late stages of COVID-19 pandemic, nurses still experienced higher levels of sleep disturbances. Those were the breadwinners of their families, volunteered to be deployed to COVID-19 wards, and more supported from family, friends, and the hospital had better sleep quality. Additionally, individuals with religious beliefs and who received support from family, friends, and the hospital demonstrated better spiritual health. It is noteworthy that working experience, sleep quality, and spiritual health influenced perceived stress factors in the late of the pandemic. In addition, this is a cross-sectional design inference that limits the ability to track the impact of changes in stress. Suggesting future longitudinal research to establish causality would enhance the discussion.

Although the world has entered a post-pandemic era, humankind has experienced a range of epidemics—from the 2002 severe acute respiratory syndrome (SARS) outbreak to the COVID-19 pandemic in 2019. Accordingly, medical staff must be prepared to face the challenges of the next major unknown infectious disease. The results of this study showed that protective equipment support from the hospital and mental support from friends and family enabled nurses to devote themselves to clinical care with peace of mind during the COVID-19 pandemic and that nurses’ spiritual needs affected their perceived stress during the late pandemic. In addition, there is a need to establish a mental health support system that can address the need of the general population. The education on coping strategies and stress management may be helpful[41]. It is recommended that medical institutions set up consultation centers for medical personnel to provide necessary psychological consultation and assistance. High-quality sleep and positive coping methods are also factors that affect stress [42], and we suggest that mindfulness is effective in improving levels of stress, sleep quality, and resilience[43]. We recommend that clinical managers use the existing experience as a reference to formulate response measures and assist frontline medical staff in facing the enormous challenges posed by infectious disease outbreaks. We also suggest that more attention be paid to the spiritual health of nurses and spiritual education programs to help frontline nurses prepare for changing and challenging clinical environments.

## Abbreviations

PSS: Perceived Stress Scale
PSQI: Pittsburgh Sleep Quality Index
SARS: Severe Acute Respiratory Syndrome
SHS-SF: Spiritual Health Scale-Short Form.
